# Transcranial direct current stimulation may modulate extinction memory in posttraumatic stress disorder

**DOI:** 10.1002/brb3.681

**Published:** 2017-04-11

**Authors:** Mascha van‘t Wout, Sharon M. Longo, Madhavi K. Reddy, Noah S. Philip, Marguerite T. Bowker, Benjamin D. Greenberg

**Affiliations:** ^1^ Department of Psychiatry and Human BehaviorAlpert Brown Medical SchoolBrown UniversityProvidenceRIUSA; ^2^ Center for Neurorestoration and NeurotechnologyProvidence VA Medical CenterProvidenceRIUSA; ^3^ Department of Psychiatry and Behavioral SciencesMcGovern Medical School at The University of Texas Health Science Center at HoustonHoustonTXUSA

**Keywords:** anxiety, brain stimulation, cognition, posttraumatic stress disorder, psychiatry, trauma, treatment

## Abstract

**Background:**

Abnormalities in fear extinction and recall are core components of posttraumatic stress disorder (PTSD). Data from animal and human studies point to a role of the ventromedial prefrontal cortex (vmPFC) in extinction learning and subsequent retention of extinction memories. Given the increasing interest in developing noninvasive brain stimulation protocols for psychopathology treatment, we piloted whether transcranial direct current stimulation (tDCS) during extinction learning, vs. during consolidation of extinction learning, might improve extinction recall in veterans with warzone‐related PTSD.

**Methods:**

Twenty‐eight veterans with PTSD completed a 2‐day Pavlovian fear conditioning, extinction, and recall paradigm. Participants received one 10‐min session of 2 mA anodal tDCS over AF3, intended to target the vmPFC. Fourteen received tDCS that started simultaneously with extinction learning onset, and the remaining 14 participants received tDCS during extinction consolidation. Normalized skin conductance reactivity (SCR) was the primary outcome measure. Linear mixed effects models were used to test for effects of tDCS on late extinction and early extinction recall 24 hr later.

**Results:**

During early recall, veterans who received tDCS during extinction consolidation showed slightly lower SCR in response to previously extinguished stimuli as compared to veterans who received tDCS simultaneous with extinction learning (*p *= .08), generating a medium effect size (Cohen's *d* = .38). There was no significant effect of tDCS on SCR during late extinction.

**Conclusions:**

These preliminary findings suggest that testing the effects of tDCS during consolidation of fear extinction may have promise as a way of enhancing extinction recall.

## 1 Introduction

Posttraumatic stress disorder (PTSD) is a disabling and often long‐term condition among returning veterans (Hoge et al., 2004; Terhakopian, Sinaii, Engel, Schnurr, & Hoge, 2008). The core deficit in PTSD has been conceptualized as pathological fear conditioning with a failure to recall extinction (Pitman, 1988; VanElzakker, Dahlgren, Davis, Dubois, & Shin, 2014). Animal models and human studies of PTSD have highlighted aberrations in neural circuitry including hyperactivity in the amygdala and dorsal anterior cingulate cortex, regions that promote fear responses, alongside hypoactivity in the ventromedial prefrontal cortex (vmPFC), a region that is thought to suppress fear responses (Milad & Quirk, 2012; Quirk, Garcia, & González‐Lima, 2006; VanElzakker et al., 2014). More specifically, vmPFC engagement during extinction learning predicts extinction success and is associated with “top‐down” modulation of amygdala‐driven fear expression (Do‐Monte, Manzano‐Nieves, Quiñones‐Laracuente, Ramos‐Medina, & Quirk, 2015; Lebrón, Milad, & Quirk, 2004; Milad et al., 2005, 2007; Phelps, Delgado, Nearing, & LeDoux, 2004; Quirk, Likhtik, Pelletier, & Paré, 2003; Rosenkranz, Moore, & Grace, 2003). Results from studies with PTSD patients revealed deficits in extinction recall (Milad et al., 2008), reduced vmPFC volume, and activation during fear extinction compared to controls (Bremner et al., 2005; Milad et al., 2009; Rauch et al., 2003; Rougemont‐Bücking et al., 2011; Shin, Rauch, & Pitman, 2006). Therefore, facilitating endogenous vmPFC activity using brain stimulation techniques, in the context of extinction learning, may be one method to improve fear extinction and retention (i.e., recall of safety memories; Milad & Quirk, 2002; Milad, Vidal‐Gonzalez, & Quirk, 2004) in those suffering from PTSD.

To this end, we recently demonstrated that applying 2 mA transcranial direct current stimulation (tDCS) for 10 min over electroencephalogram (EEG) coordinate AF3 *during* extinction learning of a previously conditioned stimulus reduced fear expression during the extinction of a second conditioned stimulus (van ‘t Wout et al., 2013). tDCS was chosen as it can modulate intrinsic neuronal activity using a weak, constant electrical current (Nitsche et al., 2008) to facilitate cognitive processing including learning and memory (Asthana et al., 2013; Coffman, Clark, & Parasuraman, 2014; Mungee et al., 2014). This work aligns with the rapidly growing body of research that indicates tDCS may have beneficial effects for psychiatric conditions associated with altered prefrontal activity or connectivity, including depression (Drevets, Price, & Furey, 2008), schizophrenia (Meyer‐Lindenberg et al., 2002), and obsessive–compulsive disorder (Chamberlain et al., 2008). For instance, recent studies have demonstrated tDCS to reduce severity of depression (Brunoni et al., 2013, 2016; Shiozawa et al., 2014) as well as promising results to reduce severity of symptoms associated with schizophrenia (Brunelin et al., 2012; Hoy, Arnold, Emonson, Daskalakis, & Fitzgerald, 2014). Yet, data on the potential effectiveness of tDCS for PTSD are limited to a small sample pilot study reporting improvement on cognitive and emotional performance after working memory training combined with tDCS (Saunders et al., 2015). Given that tDCS modulates ongoing intrinsic neuronal activity, the evaluation of tDCS combined with PTSD‐relevant emotional learning and memory processes, such as fear extinction and recall, in veterans suffering from PTSD would therefore inform its usefulness as a possible adjunct to improve existing cognitive and/or behavioral treatments for PTSD.

The goal of this feasibility study was to test whether 2 mA anodal tDCS over AF3 applied simultaneously with extinction learning processes would augment these extinction processes in veterans with warzone‐related PTSD. The selection of tDCS parameters and location was based on our prior work (van ‘t Wout et al., 2013), and our intention to target the vmPFC given its importance for extinction learning and recall. We conducted this pilot study to test the following hypotheses: (1) whether active tDCS during extinction learning compared to sham stimulation would augment late extinction learning, and (2) whether the effects of tDCS during extinction learning vs. during extinction consolidation, that is, immediately after extinction learning, on early extinction recall tested 24 hr later would differ. This idea of stimulating immediately after extinction learning, during consolidation, was based on reports that PTSD is associated with impairments in extinction recall (Garfinkel et al., 2014; Milad et al., 2008, 2009; Norrholm et al., 2011), even though extinction can be acquired. Therefore, testing the effects of tDCS during consolidation of fear extinction provides a first step to examining various possibly important time points in which noninvasive brain stimulation could be used to enhance components of extinction learning and memory (Marin & Milad, 2015).

## 2 Materials and Methods

### 2.1 Participants

Participants included 28 male combat veterans, mean age 56.25 years (*SD* = 12.3, range = 30–69 years), with a current clinical diagnosis of warzone‐related PTSD. Recruitment took place at the Providence VA Medical Center (PVAMC) by chart review and subsequent invitation if eligible and interested, as well as brochures and flyers throughout the hospital and PTSD clinic. Inclusion criteria were clinician‐based diagnosis of warzone‐related PTSD, age 18–70, male sex to avoid confounding issues of menstrual cycle, and hormonal contraceptives on fear conditioning (Glover et al., 2012; Graham & Milad, 2013; Lebron‐Milad & Milad, 2012; Milad et al., 2006; Sundström Poromaa & Gingnell, 2014; Pineles et al., 2016). Exclusion criteria were presence of any neurological/cognitive disorders, bipolar disorder, current substance abuse, or contraindication to tDCS (i.e., metal present in cranial cavity). Of the 73 individuals that were prescreened, 47 patients were ineligible and 28 patients were enrolled. All participants were on stable doses of their medication for >3 weeks prior to participation, and were asked to withhold caffeine and nicotine consumption within 2 hr before their appointment in order to minimize confounding effects of these substances on psychophysiology. The study was approved by the Brown University and PVAMC IRB and in accordance with the Declaration of Helsinki. Written informed consent was obtained after the nature of procedures was explained and prior to any study procedures.

### 2.2 Questionnaires

The PTSD Checklist for *DSM‐V* with Criterion A (PCL‐5) was administered to assess severity of self‐reported PTSD symptoms (Weathers et al., 2016). The PCL‐5 is a 20‐item self‐report scale of PTSD symptoms and is considered to have strong internal consistency, reliability, and validity (Blevins, Weathers, Davis, Witte, & Domino, 2015). The Beck Anxiety Inventory (BAI; Beck, Epstein, Brown, & Steer, 1988) and Beck Depression Inventory‐II (BDI‐II; Beck, Steer, & Brown, 1996) were used to assess self‐reported anxiety and depression. The BAI and BDI‐II are considered to have good high internal consistency and reliability (Beck et al., 1988; Dozois, Dobson, & Ahnberg, 1998). The Hoge Combat Scale was administered to characterize military combat experiences (Hoge et al., 2004). Finally, a tDCS satisfaction questionnaire was administered to quantify potential tDCS side effects and tolerability.

### 2.3 Fear‐conditioning paradigm and procedures

The experimental protocol utilized a standardized 2‐day Pavlovian fear‐conditioning–extinction‐recall paradigm (Milad, Orr, Pitman, & Rauch, 2005; Milad et al., 2005, 2007, 2008; van ‘t Wout et al., 2013) using E‐Prime 2.0 software (Psychology Software Tools, Pittsburgh, PA). Participants were presented with photographs of two different rooms, one serving as the fear acquisition context (CX+; picture of an office) and one as the fear extinction context (CX−; picture of a bookcase) in which two conditioned stimuli (CS+; red and blue light) and one never‐to‐be conditioned stimulus (CS−; yellow light) were presented. Two CS+ were included to allow comparison of results to previous studies on PTSD (Milad et al., 2009) and the effects of tDCS during fear extinction in healthy volunteers (van ‘t Wout et al., 2013), and lay the foundation for future sham‐controlled studies. For this same reason, we followed prior studies in the timing of habituation, conditioning, and extinction to occur on Day 1, and extinction recall on Day 2 approximately 24 hr later (McLaughlin et al., 2015; Milad et al., 2005, 2005, 2007, 2008; van ‘t Wout et al., 2013).

Disposable electrodes (EL503, Biopac Systems, Goleta, CA, USA) were placed over the second digits of the index and middle fingers of the dominant hand, which delivered a nonharmful electrical shock acting as unpleasant unconditioned stimulus (US). Prior to habituation, participants individually selected electric shock intensity to be “highly annoying but not painful” (Orr et al., 2000) controlled with the MP‐150 Biopac Systems using the STMISOC module. The mean shock level selected by participants was 61.1% of maximum 100 V output (*SD* = 28.3, range = 18.7–100% of maximum output) and did not differ between participants who received tDCS during vs. after extinction learning (*F*_1,27_ = 0.17, *p *=* *.68).

Skin conductance was measured using two disposable electrodes (EL507, Biopac Systems) placed on the thenar eminence of the nondominant hand. Changes in skin conductance during late extinction and early recall were the primary outcome measures. Skin conductance reflects sympathetic tone and skin conductance reactivity, that is, a change in skin conductance within a specific time window after an event can be an indication of psychological arousal and a measure of emotional, sympathetic responses (Boucsein, 2012). For that reason, skin conductance reactivity has frequently been used to assess fear and conditioning‐related responsivity (Milad & Quirk, 2012; Milad et al., 2005).

*Habituation (Day 1)*: Participants were told that the purpose of this phase was to familiarize them with all possible pictures in the experiment, and that no shock would be delivered. A total of six trials, two to be CS+ and one CS− were presented once within the CX+ and once within the CX−. The CS (+/−) and the CX (+/−) were predetermined and remained constant between participants. In all trials for each experimental phase, the CX (+/−) was presented for 9 s: 3 s alone, followed by 6 s in combination with the CS (+/−) with a 15‐s average intertrial interval (12–18 s).

*Fear conditioning (Day 1)*: Immediately prior to this phase participants were instructed that they “may or may not be shocked,” and to “pay attention to any patterns you observe between the image that you see and whether or not is followed by a shock” and that “if you observe a pattern, it will hold throughout the session and the rest of the experiment.” A total of 24 trials, 16 CS+, equally distributed among the 2 CS+, and 8 CS− trials were presented using a mirrored design so that half of all stimuli (8 CS+ and 4 CS−) occurred during the first half of conditioning and the remaining stimuli occurred during the second half of conditioning. All stimuli (CS+/−) were only depicted within the CX+. Both CS+ were paired with the US (finger shock) at a 60% reinforcement rate, resulting in 10 CS+ trials followed by the US distributed equally among both CS+. The US occurred immediately after CS+ offset and lasted 1 ms.

*Extinction learning (Day 1)*: Participants were reminded of the instructions and randomized to receive active tDCS during extinction learning (*n *= 14) or immediately after extinction learning was completed (*n *= 14). A total of 18 trials: 12 CS+, equally distributed among the 2 CS+, and 6 CS− trials were presented solely in the CX− using a mirrored design so that half of all stimuli (6 CS+ and 3 CS−) occurred to an equal degree during the first half and second half of extinction learning. No shocks were delivered. Median split determined the latter six CS+ trials as “late extinction” trials. This number of extinction trials was chosen to allow examination of tDCS augmentation on late extinction while preventing a ceiling effect on extinction learning (van ‘t Wout et al., 2013).

*Extinction recall (Day 2)*: Participants were reminded of the instructions. A total of 24 trials: 16 CS+, equally distributed among the 2 CS+, and 8 CS− using an intermixed, mirrored design within the CX− only were presented. No shocks were delivered. Early recall trials were defined by median split as the first 8 CS+ (4 red and 4 blue lights) trials.

### 2.4 Transcranial direct current stimulation

We used a neuroConn DC‐Stimulator Plus (NeuroConn, Inc., Ilmenau, Germany) in a 1 (anode) × 1 (cathode) unilateral electrode set‐up (Nasseri, Nitsche, & Ekhtiari, 2015). Active stimulation consisted of 2 mA tDCS for 10 min applied either during or immediately after extinction learning. Sham stimulation was applied for the remainder, namely after or during extinction learning, respectively. Sham stimulation consisted of 30 s of 1 mA with a ramp up and down of 30 s each. Electrodes were placed in reusable sponge pockets saturated with 0.9% normal saline and attached to the participant's skull using a rubber headband. Electrodes and sponges measured 5 × 5 cm (25 cm^2^) resulting in a 0.8 A/m^2^ current density.

**Figure 1:**
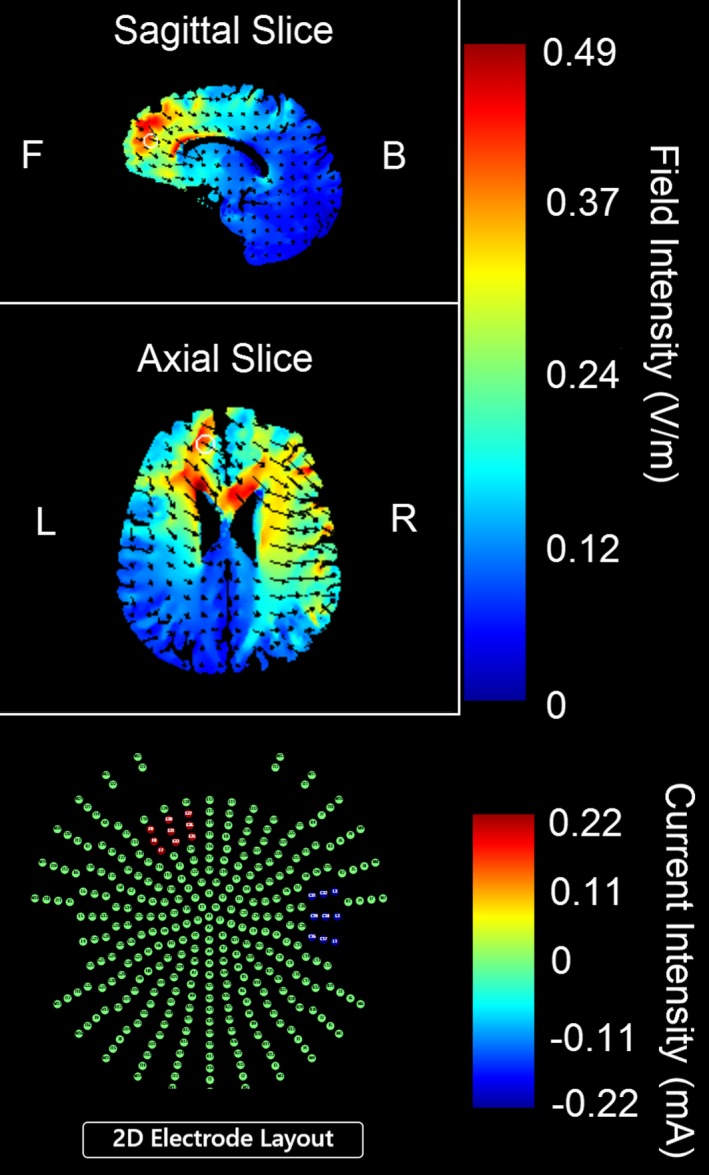
Current density modeling of 2 mA transcranial direct current based on 5 × 5 cm^2^ electrodes with the anode over the EEG coordinate AF3 and the cathode over the contralateral mastoid process using tDCS Explore neurotargeting software by SoterixMedical

The anodal electrode was placed over 10–20 EEG position AF3 and the cathodal electrode was placed over the contralateral mastoid process following our prior research in healthy volunteers (van ‘t Wout et al., 2013). This montage was chosen to deliver current to the mPFC (modeled with tDCS explore neurotargeting software by Soterix Medical, Kempe, Huang, & Parra, 2014; Figure 1) while avoiding cathodal stimulation over the prefrontal cortex. This tDCS montage is comparable to previously used montages that aimed to stimulate the vmPFC and simultaneously avoid prefrontal stimulation with the opposing electrode (Abend et al., 2016; Civai, Miniussi, & Rumiati, 2015; Zheng et al., 2016). Intensity of 2 mA was chosen to “correct” for stimulation intensity loss when electrodes are placed further away from one another (Moliadze, Antal, & Paulus, 2010), which was done to potentially reach deeper neural structures (Dmochowski, Datta, Bikson, Su, & Parra, 2011).

Placement of tDCS electrodes occurred before extinction learning on Day 1, but after conditioning. To prevent side effects, the skin under the stimulation sites was cleaned with alcohol and inspected for lesions or abnormalities; participants were instructed to notify the experimenter of any discomfort. To ensure tDCS tolerability, all participants initially received brief stimulation (1 mA for 30 s, with a ramp up/down over 30 s each). Average impedance indicating electrode contact quality during this study was 16.51 kΩ (*SD* = 6.54), well below the 55 kΩ maximum allowed by the device, and did not differ between participants who received tDCS during vs. after extinction learning, (*F*_1,27_ = 0.50, *p *=* *.82).

### 2.5 Skin conductance and statistical analyses

Biopac hardware and Biopac Acqknowledge software v.4.3 (Biopac Systems, Goleta, CA; RRID:SCR_014279) were used for skin conductance data acquisition and preprocessing. Before paradigm onset we recorded 2 min of baseline skin conductance. Participants were then asked to take a deep breath, to evaluate correct electrode attachment and conductance. Trials on which the raw skin conductance level during the presentation of the context was below 1 μS suggest inadequate data collection and were a priori removed from analyses. This resulted in the elimination of all data for one participant randomized to receive tDCS after extinction learning and between 6 and 24 trials during extinction recall for another four participants.

The raw skin conductance signal underwent a high‐ and low‐pass filter to reduce artifact. Skin conductance reactivity (SCR) for each trial was calculated by subtracting mean skin conductance level during the 3 s before CS onset (i.e., context alone was being presented) from the highest skin conductance level during the 6 s CS duration to reflect changes beyond any change produced by the presentation of context (McLaughlin et al., 2015; Milad et al., 2005, 2005, 2007; Orr et al., 2000; van ‘t Wout et al., 2013). SCR data were normalized using log transformation.

Data were analyzed using the linear mixed effects model function in SPSS (v. 20, Armon, NY; RRID:SCR_002865) to examine psychophysiological changes over individual trials while adjusting for correlations due to repeated observations within participants, following prior studies (McLaughlin et al., 2015; van ‘t Wout et al., 2013). Separate models were performed for the habituation, conditioning, extinction, and recall phase. The variables *tDCS group* (two levels: tDCS during extinction, tDCS after extinction), *stimulus type* (three levels: 2 CS+, 1 CS−), and the interaction Stimulus Type × tDCS Group were added as predictors (factors) for all experimental phases. The variable *subject* was always entered as a correlated random effects variable. A two‐sided alpha level of 0.05 was applied to determine significance in all analyses.

## 3 Results

### 3.1 Participant characteristics

Table 1 depicts a description of participant characteristics and differences between tDCS groups. For individual questionnaires with <10% of missing items, questionnaire data for that participant were included using individual mean permutation. If more than 10% of items were missing, questionnaire data for that participant were a priori not analyzed. This resulted in missing data from two participants randomized to the tDCS during extinction group on the BAI and missing data from four participants on the BDI (i.e., two in each group).

**Table 1:** Demographic and clinical characteristics of participants divided by tDCS group

Variable	tDCS during extinction	tDCS after extinction	*P*
	*M* (*SD*) or no. of participants	*M* (*SD*) or no. of participants	
Age (years)	53.36 (12.8)	59.14 (11.1)	0.21
Ethnicity	13 Caucasian, 1 African American, 0 Native American	10 Caucasian, 3 African American, 1 Native American	0.30
Education (years)	3 High school or less, 6 vocational/trade, 5 bachelor degree, 0 master degree	2 High school or less, 10 vocational/trade, 1 bachelor degree, 1 master degree	0.18
Marital Status	9 Married, 3 separated, 2 single, 0 widowed	11 Married, 0 separated, 2 single, 1 widowed	0.24
Occupation	5 Full‐time, 0 part‐time, 4 not employed, 5 retired	1 Full‐time, 2 part‐time, 3 not employed, 8 retired	0.14
Combat experience	9 Deployed once, 4 deployed ≥ 2	6 Deployed once, 5 deployed ≥ 2	0.46
HOGE (5a‐5 m)	22.69 (11.63)	22.75 (9.54)	0.98
Comorbidity	2 No comorbidity, 7 mood‐related disorder, 1 anxiety, 2 impulse control‐related disorder, 2 unknown	3 No comorbidity, 5 mood‐related disorder, 1 anxiety, 2 mood and anxiety disorder, 1 impulse control‐related disorder, 2 unknown	0.72
PCL 5	48.60 (11.1)	48.26 (10.3)	0.93
BAI	21.63 (10.9)	15.72 (7.2)	0.11
BDI‐II	20.92 (12.1)	24.33 (8.9)	0.44
Medication^a^	12 Antidepressants, 1 anxiolytics, 2 antipsychotics, 5 none	16 Antidepressants, 5 anxiolytics, 2 antipsychotics, 2 none	

HOGE, Hoge Combat Scale – higher scores reflect higher incidence of threatening combat experiences; PCL‐5, PTSD Checklist for *DSM‐V*; BAI, Beck Anxiety Inventory; BDI, Beck Depression Inventory.

a Seventeen participants used more than one type of medication.

### 3.2 Habituation

Linear mixed model results revealed a nonsignificant main effect of tDCS group (*F*_1,25.06_  = 0.76 *p *=* *.39), a borderline significant main effect of stimulus type (*F*_2,130.17_ = 2.94, *p *=* *.06), and a nonsignificant Stimulus Type × tDCS Group interaction (*F*_2,130.17_ = 1.15, *p *=* *.32). The borderline significant main effect of stimulus type was due to a tendency of one future CS+ to be associated with a larger skin conductance magnitude (*M* = 0.57) as compared to the other future CS+ (*M* = 0.52) and CS− (*M* = 0.54). This finding was likely due to an orienting response on the first trial, and removal of the first trial from the analyses resulted in a nonsignificant main effect of stimulus type (*F*_2,104_ = 0.71, *p* = .50). The main effect of tDCS group as well as the Stimulus Type × tDCS Group interaction remained nonsignificant (*F*_1,26.99_ = 0.51, *p *=* *.48 and *F*_2,104_ = 1.66, *p* = .19), respectively.

### 3.3 Fear conditioning

**Figure 2:**
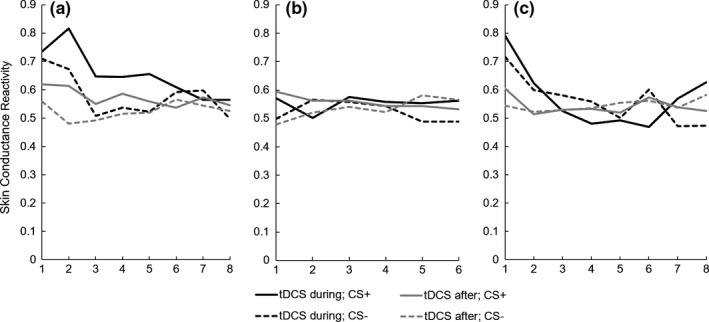
Normalized skin conductance values (in μS) for CS+ and CS− trials over time for conditioning (a), extinction (b), and recall (c) separated by tDCS group

The first trial was omitted from analyses to account for an orienting response (Mungee, Burger, & Bajbouj, 2016; Mungee et al., 2014; van ‘t Wout et al., 2013). Before discarding any data, adequacy of conditioning was examined as defined by CS+ > CS− during conditioning, and CS+ during conditioning >CS+ during habituation (Asthana et al., 2013; Mungee et al., 2014, 2016; Phelps et al., 2004; van ‘t Wout et al., 2013). Following this definition, five participants did not condition appropriately and were removed from subsequent analyses. In the remaining sample, adequate conditioning was obtained since: (1) a linear mixed model comparing SCR to CS+ during habituation vs. conditioning demonstrated a main effect of experimental phase (mean CS+ during conditioning: 0.66 > mean CS+ during habituation: 0.55; *F*_1,417_ = 5.91, *p *=* *.02), and (2) a linear mixed model examining SCR to CS+ and CS− during conditioning, revealed a significant main effect of stimulus type (CS+ during conditioning: 0.60 and 0.59 > CS− during conditioning: 0.55; *F*_2,480_ = 4.36; *p *=* *.01). We further observed a nonsignificant main effect of tDCS group (*F*_1,20_ = 1.60 *p *=* *.22) and a nonsignificant Stimulus Type × tDCS Group interaction (*F*_2,480_ = 1.38, *p *=* *.25). This suggests that participants in both groups (tDCS during or after extinction) conditioned comparably and appropriately (Figure 2a).

### 3.4 Late extinction

A linear mixed model on late extinction trials resulted in a nonsignificant main effect of Stimulus Type (CS+: 0.54; CS+: 0.56; CS−: 0.53; *F*_2,172_ = 0.598; *p *=* *.55), a nonsignificant main effect of tDCS group (all CS combined with tDCS during extinction: 0.54; all CS combined with sham during extinction: 0.55; *F*_1,20_ = 0.01; *p *=* *.91), and a nonsignificant Stimulus Type × tDCS Group interaction (*F*_2,172_ = 1.34, *p *=* *.26). The comparable SCR to either CS+ as compared to the CS− during late extinction training indicate the occurrence of extinction, which was similarly effective for those who received tDCS during this phase vs. those who received sham stimulation during this phase (but who would continue to receive tDCS immediately afterward; Figure 2b).

### 3.5 Early recall

Linear mixed model results on early recall trials demonstrated a nonsignificant main effect of stimulus type (*F*_2,220.7_ = 0.42, *p *=* *.66), a trend toward a significant main effect of tDCS group (*F*_1,20.2_ = 3.42, *p *=* *.079), and a nonsignificant Stimulus Type × tDCS Group interaction (*F*_2,220.7_ = 0.35, *p *=* *.71). Given our focus on the main effects of tDCS during early extinction recall, we explored this trend toward significance in early CS+ only recall trials. A linear mixed model confirmed the borderline significant main effect of tDCS group (CS+ combined with tDCS during extinction: 0.62; CS+ combined with tDCS after extinction: 0.54; *F*_1,19,04_ = 3.12; *p *=* *.08; Cohen's *d = *.38). This suggests that participants who received tDCS immediately after extinction had slightly lower SCR to CS+ across early recall trials than those who received tDCS during extinction learning, generating a medium effect size due to small sample size (Figure 2c).

### 3.6 Tolerability of tDCS

Stimulation was well tolerated. Besides expected temporary stimulation site erythema, no adverse events or discomfort were reported. Of all participants, 18/28 (64.3%) reported being satisfied, 9 participants were unaffected, and 1 participant was slightly dissatisfied attributed to lack of clinical change. There was no significant difference in satisfaction between groups, χ^2^(2) = 1.11, *p *=* *.57.

## 4 Discussion

The results of this pilot study show that veterans with warzone‐related PTSD who received tDCS during extinction consolidation demonstrated moderately better extinction memory during extinction recall than those who received tDCS during extinction learning. However, veterans who received tDCS during extinction showed similar adequate extinction learning as veterans who received sham stimulation at that time, evidenced by the nonsignificant differences between tDCS groups and the two previously conditioned stimuli vs. the never conditioned stimulus during late extinction. This absence of a tDCS effect on extinction learning is consistent with previous studies (Abend et al., 2016; van ‘t Wout et al., 2013) and suggests that tDCS does not instantaneously influences fear‐based responses.

There are several potential explanations for our results. One is that tDCS allowed modulation, albeit small, during a window of opportunity immediately after fear extinction learning on extinction consolidation when the extinction memory is still in a short‐term, labile state (McGaugh, 2000). Support for this idea can be found in animal studies where inactivation of the rodent vmPFC immediately after extinction training impaired memory for extinction (Burgos‐Robles, Vidal‐Gonzalez, Santini, & Quirk, 2007; Hikind & Maroun, 2008; Laurent & Westbrook, 2008; Sotres‐Bayon, Diaz‐Mataix, Bush, & LeDoux, 2009). Furthermore, extinction success is associated with the amount of high frequency bursting in rodent vmPFC neurons during (Chang, Berke, & Maren, 2010) as well as after extinction training (Burgos‐Robles et al., 2007). Applied to our findings, tDCS immediately following extinction learning may have augmented extinction consolidation, and moderately improved extinction memory observed by slightly lower SCR during early extinction recall. This provides modest initial support for the suggestion that noninvasive neuromodulation during consolidation of safety memories might be a worthwhile direction for further investigation to optimize tDCS protocols as an adjunct to exposure‐based psychotherapy for PTSD (Marin, Camprodon, Dougherty, & Milad, 2014; Marin & Milad, 2015).

Another possible explanation may be that tDCS during extinction learning may have somewhat worsened extinction memory, resulting in slightly higher SCR during early recall, compared to tDCS after extinction learning. On this view, stimulation may interfere with extinction learning and/or consolidation, thereby generating a less effective extinction memory trace to inhibit fear expression during early extinction recall. For instance, the possibility of worsening extinction memory by tDCS during extinction learning has recently been reported (Abend et al., 2016). Abend et al. (2016) observed that 1.5 mA anodal tDCS for 20 min targeting the mPFC during fear extinction resulted in an overgeneralization of fear, demonstrated by comparably high SCR to both CS+ and CS− during extinction recall. It is interesting to note that we also observed comparable SCR to CS+ and CS− during early recall trials, with a trend toward higher SCR for both CS+/− in veterans who received tDCS during extinction as compared to veterans who received tDCS after extinction (Abend et al., 2016). This further points to the complexity of timing of electrical stimulation in relation to extinction learning in humans (Abend et al., 2016), an observation that was also recognized by Milad et al. (2004) in rodents.

Finally, it is possible that both tDCS conditions (i.e., tDCS during and after extinction training) may have influenced subsequent recall, with veterans who received tDCS following extinction training showing a slightly larger beneficial effect. Lack of a third control group who received sham stimulation only is an important limitation of our study. Our data are therefore of heuristic value and needs to be interpreted cautiously. Nonetheless, this study highlights the feasibility, tolerability, and potential ability of tDCS combined with fear extinction‐related processes to modulate memory for extinction in a sample of veterans with PTSD. Furthermore, this study points to a possibly novel time point, that is, extinction consolidation, that has not been previously tested, but during which tDCS may modulate memory for extinction.

Some design aspects are worth noting; in our experimental paradigm, extinction training was initiated within approximately 10 min after fear conditioning, which was done in order to allow comparison to previous studies (McLaughlin et al., 2015; Milad et al., 2005, 2005, 2007, 2008; van ‘t Wout et al., 2013). Prior research supports the presence of differences in extinction processes depending on their temporal relationship with fear conditioning (Myers, Ressler, & Davis, 2006). Specifically, extinction in close temporal proximity to conditioning, as done in our study, may promote fear “unlearning” or fear memory erasure, instead of additional extinction memory formation (Myers et al., 2006), with the latter being more applicable to PTSD. Of further relevance to PTSD is that the stimuli used in this Pavlovian fear‐conditioning paradigm were trauma neutral. The effects of tDCS may be different when applied in combination with habituation‐like processes to trauma‐relevant cues using script‐driven or exposure therapy for PTSD, or if we had increased the time between conditioning and extinction to 24 hr. Moreover, we did not examine longer lasting effect of tDCS or whether tDCS impacted fear recovery processes such as renewal or reinstatement. These are important questions that should be addressed in future studies to further determine the potential for tDCS as an adjunct to PTSD treatment.

Specific to tDCS parameters and montage, we utilized our previously used tDCS settings that modulated fear extinction in healthy volunteers (van ‘t Wout et al., 2013). This protocol was designed to deliver current to the vmPFC and avoid cathodal effects on the prefrontal cortex. However, other brain regions involved in fear processing (i.e., the dorsolateral prefrontal cortex, Mungee et al., 2014; Asthana et al., 2013; Delgado, Nearing, LeDoux, & Phelps, 2008; and anterior cingulate cortex, Etkin, Egner, & Kalisch, 2011; Milad et al., 2007) may have been inadvertently stimulated. Also, tDCS was applied once for a duration of 10 min total and effects of tDCS may be expected to increase with greater duration and number of sessions (Brunoni et al., 2016). Moreover, we cannot rule out that veterans who received tDCS during extinction learning may have experienced nonlinear after effects during immediate consolidation, possibly hindering consolidation and subsequent recall. While prior studies demonstrated the after effects of tDCS are typically in the direction of stimulation (Nitsche & Paulus, 2000, 2001), others have suggested that the application of 2 mA stimulation intensity—done here in order to “correct” for greater distance between electrodes to reach deeper brain regions (Dmochowski et al., 2011; Moliadze et al., 2010)—may result in nonlinear after effects that are opposite from the direction during stimulation (Amadi, Allman, Johansen‐Berg, & Stagg, 2015; Batsikadze, Moliadze, Paulus, Kuo, & Nitsche, 2013). However, these studies on tDCS after effects focused on the motor cortex, and as a result there is uncertainty about the direction of polarity after effects of prefrontal tDCS. The complexity of ultimate polarity effects of tDCS is in fact inherent to the nature of tDCS, in which current passes through all tissue in between the surface electrodes (Bikson et al., 2016; Philip et al., 2017), as demonstrated by electrical field modeling (Datta et al., 2009).

Despite current unknowns and general limitations of tDCS, the main limitations of this pilot study are the absence of a PTSD control group that received sham stimulation only, restriction to males, and small sample size; all of these factors raise questions about statistical power. Other limitations involve the possibility that our fear‐conditioning paradigm did not elicit strong conditioned responses, as well as the inability to determine possible interactions between tDCS, fear extinction processes, and psychotropic medication use in our sample, as these could influence the (after)effects of tDCS (Liebetanz, Nitsche, Tergau, & Paulus, 2002; Monte‐Silva et al., 2009; Nitsche et al., 2006, 2009). However, the focus of this pilot study was on the feasibility of combining tDCS with a PTSD‐relevant emotional learning paradigm in a real‐world clinical sample, to guide subsequent clinical research. From that perspective, our study provides a first step to examining various potential important time points during which noninvasive brain stimulation, and tDCS in particular, could be used to enhance fear extinction memory processes. Future research will need to replicate these findings in a larger, more diverse sample with an adequate control condition to determine the effects of tDCS‐modulated consolidation of fear extinction.

## 5 Conclusion

To our knowledge, this is the first study to evaluate whether tDCS may augment fear extinction and recall in veterans with warzone‐related PTSD. Our observation that tDCS applied during immediate extinction consolidation may have some influence on extinction recall in veterans with PTSD is encouraging for future research to further define and narrow the parameter space for hypothesis‐driven testing of the potential for tDCS to augment fear extinction‐related processes for ultimate clinical application.

## Conflicts of Interest

B. D. G. discloses support in part by the NIH Grant P50 MH106435 and the Department of Veterans Affairs, Veterans Health Administration, Office of Research and Development, Rehabilitation Research and Development Service Project N9228‐C. M. vt W. discloses support by the Department of Veterans Affairs, Veterans Health Administration, Office of Research and Development, Rehabilitation Research and Development Service Project N9228‐C. N. S. P. is supported by Department of Veterans Affairs grant IK2 CX000724.
